# College and Hospital Notes

**Published:** 1907-01

**Authors:** 


### COLLEGE AND HOSPITAL NOTES.

The Woman's Hospital of New York, established by Dr. J. Marion Sims, has moved into its new building on Cathedral Heights, just below the uncompleted cathedral of St. John the Divine. The opening of this splendid new structure, which stands seven stories high, was celebrated by appropriate exercises recently, conducted under the auspices of Bishop Henry C. Potter, who offered prayer, after which John E. Parsons, president of the board of trustees, made a brief address recounting the incidents connected with the change of location.

Dr. D. B. St. John Roosa was the next speaker; he made reference during the course of his address to the methods adopted in Pennsylvania where the state contributes to the support of hospitals. He contrasted the conditions prevailing in this country with those in vogue in England. For example, the result may be seen in London which is placarded with appeals to help support the hospitals and reminders to the public that English hospitals are maintained by popular subscription. England, he said, has a theory, shared by no other nation, that government support of hospitals is not wise. He also warned against the future possibility of diverting this hospital from its original purpose; that is, from a hospital strictly for the treatment of diseases of women into more general uses.

General Horace Porter also spoke, giving an address full of reminiscences of the battlefield and of what women had done there in caring for the wounded.

At the annual meeting of the Buffalo General Hospital Association, held at the hospital on High street, November 25, 1906, seven trustees were elected for the ensuing year. The new board of trustees met immediately and elected officers as follows: Charles W. Pardee, president; Henry M. Watson, vice-president;

H. Edson Webster, secretary; Elliott C. McDougal, treasurer.

Scatcherd Hall in the main building of the hospital was dedicated in memory of James N. Scatcherd, Thanksgiving day, November 29, 1906. The hall was presented to the hospital by the late Mr. Scatcherd's son, John N. Scatcherd. The space occupied by Scatcherd Hall was formerly the old clinic. It has been rebuilt into a modern and beautiful lecture-room which will seat about 150 persons.

President Pardee, who presided, made appropriate remarks introducing Mr. Scatcherd, who said:

For a long time I had been looking for some way to do something for this institution that would be in memory of my father. A little while ago Mr. Pardee told me that it was the intention of the trustees to convert this room into a chapel, and I at once saw that it was the very opportunity I had been looking for. Plans were submitted, and the room you are in today is the result.

I think it is particularly appropriate to dedicate it on this Thanksgiving Day, bright and sunny as it is, and I want to say this is the happiest Thanksgiving Day of my life. It is my sincere hope that everyone who enters here will be benefited and made stronger by the services which will be heard inside.

---

It is announced from Washington that bids will be invited for the new Marine Hospital, at Buffalo, on or about February 1, 1907. It is expected that the work of construction will begin in the early spring.

